# Enabling accelerated vaccine roll-out for Public Health Emergencies of International Concern (PHEICs): Novel Oral Polio Vaccine type 2 (nOPV2) experience^☆^

**DOI:** 10.1016/j.vaccine.2022.02.050

**Published:** 2023-04-06

**Authors:** Grace R. Macklin, Corey Peak, Martin Eisenhawer, Feyrouz Kurji, Ondrej Mach, John Konz, Chris Gast, Novilia Sjafri Bachtiar, Ananda S. Bandyopadhyay, Simona Zipursky, on behalf of the nOPV2 Working Group

**Affiliations:** a London School of Hygiene and Tropical Medicine, London, United Kingdom; b Polio Eradication, World Health Organisation, Geneva, Switzerland; c Bill and Melinda Gates Foundation, Seattle, Washington, United States; d FDK Consulting LLC, Kirkland, Washington, United States; e PATH, Seattle, Washington, United States; f Bio Farma, Bandung, Indonesia

**Keywords:** Poliovirus, Vaccine, Emergency use listing, Novel oral poliovirus vaccine, Public health emergency of international concern

## Abstract

To address the evolving risk of circulating vaccine-derived poliovirus type 2 (cVDPV2), Global Polio Eradication Initiative (GPEI) partners are working closely with countries to deploy an additional innovative tool for outbreak response – novel oral polio vaccine type 2 (nOPV2). The World Health Organization’s (WHO) Prequalification program issued an Emergency Use Listing (EUL) recommendation for nOPV2 on 13 November 2020. The WHO’s EUL procedure was created to assess and list unlicensed vaccines, therapeutics and diagnostics to enable their use in response to a Public Health Emergency of International Concern (PHEIC). nOPV2 was the first vaccine to receive an EUL, paving the way for other emergency vaccines. In this report, we summarise the pathway for nOPV2 roll-out under EUL.

## 1 Introduction

Whilst the wild-type poliovirus type 2 (WPV2) has been eradicated, the surge of circulating vaccine-derived poliovirus type 2 (cVDPV2) outbreaks is considered to be a major a challenge for the Global Polio Eradication Initiative (GPEI). In 2020, there were 1062 cVDPV2 paralytic poliomyelitis cases, with detections reported from 30 countries across four World Health Organization (WHO) regions - African, Eastern-Mediterranean, Western-Pacific and European regions.

The current Sabin oral poliovirus vaccine (OPV) strains can lose their attenuating mutations over time, particularly when transmitted from person-to-person [1]. Rarely, this results in vaccine-associated paralytic poliomyelitis (VAPP) in vaccine recipients and close contacts, or generation of vaccine-derived polioviruses (VDPV) with transmissibility and neurovirulence characteristics similar to wild poliovirus. In settings of low population immunity, VDPVs can persist in the community and result in outbreaks of circulating VDPVs (cVDPVs) [2]. The strategy of responding to cVDPV2 with monovalent oral poliovirus vaccine type 2 (mOPV2) has been largely successful in stopping transmission of cVDPV2; however, due to the genetic instability described above and waning population immunity following the global cessation of routine use of type 2 Sabin OPV, an increasing number of new cVDPV2 outbreaks are attributable to mOPV2 use [3]. While inactivated poliovirus vaccine (IPV) used in routine immunization and occasionally in outbreak response protects against paralytic disease from type 2 polioviruses, it provides limited primary intestinal mucosal immunity necessary to stop outbreaks of cVDPV2 or prevent their emergence following type 2 Sabin OPV use [4].

A central priority of GPEI is to develop oral poliovirus vaccine strains that are more genetically stable than Sabin OPV [5]. The novel oral polio vaccine type 2 (nOPV2) strains are modified versions of the Sabin mOPV2 with enhanced genetic stability [6], [7]. Therefore, nOPV2 is anticipated to have a significantly reduced risk of evolution to a VDPV compared to the existing mOPV2.

In 2019, the GPEI established an nOPV2 Working Group to oversee the multifaceted approach for delivering a vaccine for a Public Health Emergency of International Concern (PHEIC) (Box 1). On 13 November 2020, nOPV2 received a recommendation for use under WHO Emergency Use Listing (EUL) and was first used as part of outbreak response in Nigeria on 13 March 2021. In this paper, we summarise the accelerated pathway for clinical development, manufacturing and programmatic introduction of nOPV2.

Box 1 **Overview of the nOPV2 Working Group.**

The nOPV2 Working Group is a time-limited group established to manage and coordinate across Global Polio Eradication Initiative (GPEI) activities to enable a rapid and effective rollout of nOPV2 as the tool of choice for responding to cVDPV2 outbreaks.

The core nOPV2 working group is composed of representatives from all six GPEI partner agencies (Rotary International, UNICEF, Bill and Melinda Gates Foundation (BMGF), Gavi, the Vaccine Alliance (GAVI), US Centers for Disease Control and Prevention (US-CDC), and World Health Organisation (WHO).

In order to advance work in a several key technical areas, a collection of specific sub-groups that include membership from experts beyond GPEI were established at different time-points: **Research, Data Analysis and Modelling; Initial Use Country Support**; **Manufacturer Support** (including regulatory support); **Genetic Characterisation; Safety;** and nOPV2 WG liaisons for vaccine supply, communications and readiness verification.

In addition, the core nOPV2 working group oversees policy development for nOPV2 and co-ordinates with two independent advisory boards: WHO Strategic Advisory Group of Experts on Immunisation (SAGE) and the Global Advisory Committee on Vaccine Safety (GACVS). In 2021, support for development of nOPV1 and nOPV3 vaccine candidates was also included in the working group’s expanded focus areas, transitioning it to be renamed as “nOPV Working Group”.

## 2 Pre-clinical development

In 2011, a consortium was formed with funding support from the Bill & Melinda Gates Foundation to develop improved OPV virus strains, with the vision that a collaborative effort using a combination of strategies would have the greatest chance of success. Researchers from the National Institute for Biological Standards and Controls (NIBSC), the US Centers for Disease Control and Prevention (US-CDC), the US Food and Drug Administration (FDA), and the University of California at San Francisco collaborated to design, produce and test several novel OPV strains in a variety of pre-clinical studies. The strains were assessed through intraspinal inoculation in a transgenic mouse neurovirulence model; passaging in cell culture under selective pressure conditions known to lead to reversion of Sabin (such as 37 degrees Celsius in Vero Cells) followed by deep sequencing; and infectious yield measurements. This identified candidates that were at least as attenuated as Sabin type-2 strains, had enhanced genetic stability (reduced potential to revert to a neurovirulent phenotype), and similar antigenicity and immunogenicity [6], [7].

Two candidate nOPV2 strains, referred to as nOPV2-c1 and –c2, were selected based on these pre-clinical studies to take forward to clinical trials [6], [7]. The candidates used different combinations of 5 modifications of the Sabin-2 genome, including changes to the ribonucleic acid (RNA) sequence in the 5′ untranslated region of the polio genome (5′ UTR), the capsid protein coding region (P1), the non-structural protein 2C, and the polymerase 3D [6], [7]. Full details of the genetic modifications and their purposes are summarized in Table 1.

**Table 1:** Genetic modifications of candidate 1 (c1) and candidate 2 (c2) novel oral poliovirus vaccine type 2 (nOPV2).

Modification	c1*	c2**	Purpose
S15 domain V changes	X	X	• Improved stability of attenuated phenotype. Specifically, improve genetic stability of the domain V attenuating mutation to avoid reversion by single nucleotide changes.
cre relocation	X		• Reduce frequency of recombination events. Specifically, a single recombination event replacing domain V will also remove cre, making virus non-viable and non-infectious.
Polymerase (HiFi):Higher Fidelity changes	X		• Improved stability of attenuated phenotype. Specifically, improved fidelity of replication leading to less genetic drift and reversion.
Polymerase (Rec) changes	X		• Reduce frequency of recombination events thereby reducing ability of population to improve replication fitness.
Capsid P1 region codon deoptimization		X	• Improved stability of attenuated phenotype. • May also reduce transmission (less infectious per particle). • May enhance innate immune response against vaccine. • May increase attenuation.

*Candidate 1 (S2/cre5/S15domV/rec1/hifi3). Strain selected for EUL application.

**Candidate 2 (S2/S15domV/CpG40).

## 3 Clinical development

**Fig. 1:**
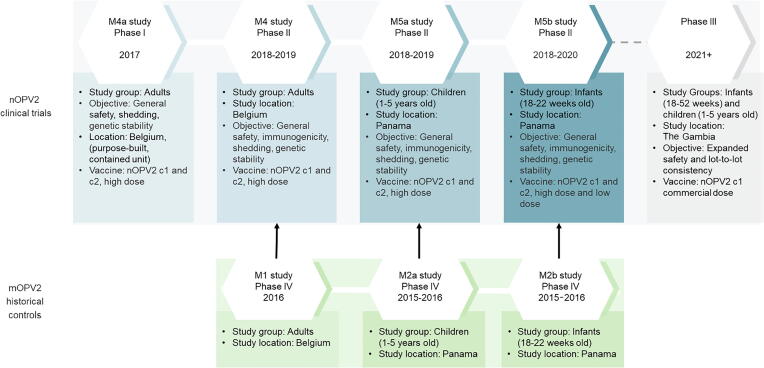
nOPV2 Clinical Development Plan. Additional phase II studies and control trials that were not prioritised for EUL data submission are not shown on this figure and are described in the text.

The clinical development for both nOPV2-c1 and –c2 investigated the safety, immunogenicity, shedding and genetic stability (less reversion to neurovirulence) of the candidates in Phase I and Phase II trials, in Belgium and Panama. A summary of the nOPV2 trials and historical control trials is provided in Fig. 1. Substantial efforts were made to accelerate the clinical development of nOPV2, which are summarised in Box 2.

Box 2 **Summary of clinical development acceleration methods.**

1. Implementing five historical control trials in approximately 6 months’ time in advance of global cessation of Sabin OPV2 use, to generate comparator data
2. Executing nOPV2 clinical trials in staggered, parallel trials (e.g. age descension from adults to toddlers to infants in the phase II studies)
3. Studying only a high-dose level in participants who have been fully vaccinated against all polio types.
4. Empowering a data and safety monitoring board (DSMB), common to all nOPV2 studies, with decision rights regarding age de-escalation and dose escalation while trials were on-going
5. Using satellite sites for rapid subject enrollment, and real-time data generation by primary lab to inform trial conduct
6. Generating multiple incremental interim trial reports to enable rolling EUL submission and review
7. Major scale up and optimization of laboratory capacity to generate data for EUL submission, with 20,000 stool samples and 5,000 serological samples tested by US centers for Disease Control and Prevention (US-CDC).

Phase IV historical control studies were conducted in Panama and Belgium to provide baseline data with mOPV2 before the global withdrawal of type 2-containing OPV in May 2016. As the nOPV2 candidates were not yet available for clinical trials, these were conducted subsequently: to maximize comparability of data, the mOPV2 phase IV trials were designed to parallel the expected design of the phase I and II nOPV2 studies.

The phase I first-in-human study with nOPV2 was conducted in Belgium: 30 healthy adults (aged 18–50 years) previously immunized exclusively with IPV were administered a single dose of nOPV2-c1 or nOPV2-c2 vaccine and isolated for 28 days in a purpose-built containment facility [8], [9]. This study provided an initial demonstration of vaccine safety, viral shedding and genetic stability: this allowed progression into the larger phase II study, with administration to non-IPV vaccinated individuals, and was influential in the WHO Containment Advisory Committee recommendation that subsequent studies could be done outside of containment. Additionally, evaluation of intestinal and serum neutralizing antibodies after a single dose of nOPV2 at dose 10^6^ CCID_50_ (50% cell culture infectious dose) was also conducted [8], [10].

The subsequent, larger, phase II study was conducted in Belgium with 200 previously OPV-vaccinated healthy adults assigned to receive one or two doses of nOPV2-c1 or nOPV2-c2; a further 50 participants, previously vaccinated with IPV, were assigned to nOPV2-c1 nOPV2-c2 or placebo [11]. The results demonstrated safety in a larger group of adults and supported the assessment of the vaccine candidates in children and infants.

Two phase II studies were conducted in Panama: one in children between 1 and 5 years of age that had received prior trivalent oral polio vaccine (tOPV) and/or IPV-vaccination, and the second in infants aged 18 weeks that had previously received bOPV and a single dose of IPV [12]. The immunogenicity of nOPV2-c1 and nOPV2-c2 was evaluated at low and high dose potencies in these phase II studies (10^5^ CCID_50_ and 10^6^ CCID_50_ [high dose, HD]) [11], [12].

Data from the clinical studies show both nOPV2 candidates to be well-tolerated in adults, young children, and infants, with no specific safety concerns identified [8], [11], [12]. There have been no serious adverse events considered to be related to vaccination with nOPV2. The most important immunogenicity evaluation was the seroprotection rate and seroconversion rate, 28 days following a single dose, in 18–22-week-old infants. The primary immunogenicity hypothesis of non-inferiority of seroprotection rate to mOPV2 was met for nOPV2-c1 at both the high and low doses; however, it was only met for nOPV2-c2 at the high dose [12].

Additional studies are underway in Bangladesh: a trial in polio-vaccine naive neonates and a concomitant bivalent oral polio vaccine (bOPV)-nOPV2 administration study. In addition, a phase III safety and lot-to-lot consistency trial has been initiated in The Gambia. These studies will further expand the clinical safety database in the target population (children aged 0–5 years old).

**Fig. 2:**
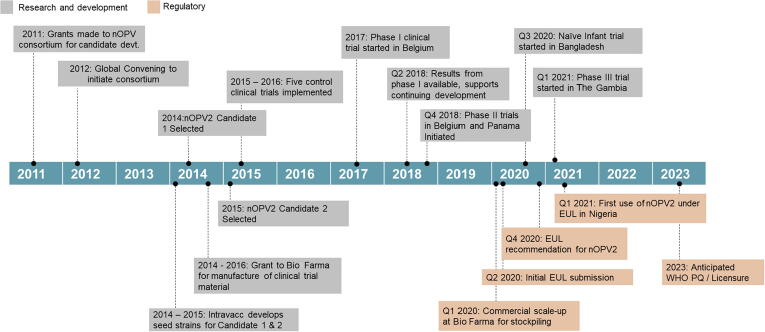
Timeline of nOPV2 development.

All through the clinical development process, several unprecedented challenges were encountered, and innovative mitigation strategies were applied to overcome these issues in a timely manner. Conducting clinical evaluation of a strain of poliovirus that was under containment introduced multiple complexities in the development process, including the insertion of a phase I study under fully contained conditions, and conducting near real-time laboratory and clinical evaluation to inform decision making on subsequent studies without containment. Evaluation of unique endpoints, such as pattern of reversions in key areas of vaccine virus genome and neurovirulence in modified transgenic mice assays necessitated a series of consultations and extensive engagement of technical authorities to ensure the phase II studies were designed in a way that would inform public health and regulatory decision making, based on the unique epidemiologic context. Precedence and prior positioning on some of these factors could have contributed to a further accelerated development process (see Fig. 2).

## 4 Down-selection of candidates and manufacturing

In 2019, with the cVDPV2 situation worsening and resulting in an urgent need for nOPV2, a decision was made to move forward with at-risk, at-scale production of nOPV2, based on a review of the available clinical and manufacturing data. Based primarily on manufacturing yield information, one of the two candidates - nOPV2 c1 - was prioritised over c2, driven by the projected program need of large number of doses at the earliest time-frame. The low-dose 10^5^ CCID^50^ of either candidate would allow faster scale up of production, however, initial data suggested nOPV2-c1 would have higher potency at that dose - the high-dose formulation that would likely be required for nOPV2-c2 would preclude sufficient production to meet the epidemiological need. The decision to proceed with nOPV2-c1 was confirmed from subsequent data from the Phase II trial in Panama, which became available in early 2020, demonstrating non-inferiority of immunogenicity of the low dose formulation for c1 but not nOPV2-c2 [12].

The vaccine manufacturer, PT Bio Farma, committed to produce up to 200 million doses of nOPV2 by the end of 2020 to enable the vaccine to be deployed when WHO issued an emergency recommendation for use. Having a relatively large number of doses available in this timeframe was critical as the vaccine would potentially be needed for outbreak response campaigns of national scale. Bulk and finished product manufacturing was first performed at a pilot plant at BioFarma and then later shifted to commercial facilities, which received regulatory inspections from WHO Prequalification (PQ) inspection team as well as from the Indonesian National Regulatory Authority (Badan POM) to ensure compliance with international good manufacturing practices (GMP) standards. The finished product from the pilot plant was filled in 20 dose vials and is available for study purposes. To maximize production capacity for nOPV2 for field use, a decision was taken to produce the vaccine in the commercial facility in 50 dose vials, which is the presentation that received WHO EUL.

## 5 Use of nOPV2 under Emergency Use Listing (EUL)

The WHO PQ team developed the EUL process to expedite the availability of unlicensed medical products needed in public health emergency situations and to assist interested UN procurement agencies and Member States in determining the acceptability of using specific products in the context of a public health emergency of international concern (PHEIC) [13]. The EUL procedure involves a rigorous risk-based assessment by an expert advisory committee based on an essential set of available data on quality, safety, and efficacy/immunogenicity/performance [13].

Considering the cVDPV2 outbreaks and continued declaration of polio as a PHEIC, WHO and the Badan POM agreed to focus on potential use of nOPV2 (c1) under an EUL [14]. Without EUL, nOPV2 would not be available until at least 2023, due to the timelines for pre-qualification and licensure of vaccines, including requirement of phase III clinical data. Pre-alignment discussions took place between the nOPV2 development team, Badan POM and WHO PQ, which were necessary to align expectations and provide early feedback. It was agreed that an EUL assessment could be undertaken once sufficient data in young children and infants from phase II studies became available and that a rolling submission and evaluation of clinical data would be used, with data being shared as it becomes available; subsequentially, a roadmap was published for the evaluation of nOPV2 under EUL [15].

A positive recommendation for use of nOPV2 under EUL was made on November 13, 2020, with nOPV2 becoming the first vaccine to receive an EUL recommendation from the WHO. Should data from field use further support vaccine safety and effectiveness, nOPV2 use would continue under the EUL until the clinical data are available to support licensure and WHO prequalification of nOPV2.

For vaccines listed under an EUL, post-deployment monitoring measures may be required [13]. For nOPV2, these measures include monitoring and analysis of the safety, genetic stability and effectiveness of the vaccine. Any country wishing to deploy nOPV2 under the EUL must meet a set of pre-defined readiness verification criteria, which are assessed by global and regional teams, to ensure they are ready to implement these measures, and are prepared to respond to any unanticipated findings.

## 6 Programmatic roll-out: A phased approach

The GPEI have developed a phased approach to the roll-out of nOPV2 under EUL, which was endorsed by WHO Strategic Advisory Group of Experts on Immunization (SAGE). As nOPV2 had never been used outside clinical trials, a strict set of criteria were developed for the first uses of nOPV2, in addition to the mandatory post-deployment monitoring requirements of EUL (Box 3). The initial use period was expected to last for approximately six months. Given that cVDPV2 outbreaks disproportionately affect areas with weaker healthcare systems and inaccessible areas, these criteria were designed to ensure close monitoring of vaccine safety and performance, and the ability to detect any unanticipated events and respond to these quickly and effectively to minimize risk and impact on broader immunization activities.

Box 3 **Criteria for the initial use of nOPV2 under EUL, as endorsed by SAGE.**

Essential criteria:

1. Detection of disease or virus (VDPV2 detection).
2. Capacity to acquire and distribute the vaccine during outbreak response in a timely manner (including accessibility to population and healthcare system).
3. Capacity to respond to an unanticipated finding in a way that minimises risk and impact on the broader immunisation programme (adverse events, vaccine acceptance amongst the population).
4. Adequate surveillance to monitor vaccine behavior (safety and genetic stability) and disease incidence. Specifically, for nOPV2, this includes:
  a. Adverse Event Following Immunization (AEFI) and Adverse Event of Special Interest (AESI) surveillance.
  b. Acute flaccid paralysis (AFP) surveillance.
  c. Environmental surveillance (ES)
5. At least 12 weeks from type 2 containing Sabin OPV (mOPV2, tOPV) campaign in the same area.

Recommended criteria:

6. At least 6 weeks from bOPV campaigns in the same area.

The proposed intervals between nOPV2 use and other Sabin OPV (tOPV, mOPV2 or bOPV) campaigns during the initial use phase were in place to minimize confounding in assessment of effectiveness of nOPV2 and reduce overlap of any safety signals associated with different vaccines. In addition, this time separation would also reduce the risk of genetic recombination between vaccine viruses. The time period was based on the duration of transmission of Sabin-strain vaccine in the community after a vaccine campaign and duration between exposure and onset of adverse events [16], [17]. Routine immunization with bOPV will be un-interrupted for infants, as the level of vaccine virus circulating in the population is substantially lower from routine immunization than following a vaccination campaign (such as national immunization days and outbreak response) [18].

The Global Advisory Committee on Vaccine Safety (GACVS) established a sub-committee on nOPV2 to provide an independent assessment of safety data generated following use of nOPV2 under the EUL. After provision and review of vaccine safety, the initial use criteria was removed in October 2021, enabling nOPV2 to become the vaccine of choice to respond to cVDPV2 outbreaks, as endorsed by SAGE [19], [20]. However, countries must still meet the post-deployment monitoring requirements outlined by the EUL until full licensure and WHO prequalification of nOPV2.

## 7 Programmatic roll-out: Preparing countries

Any country planning to use nOPV2 under EUL must have in place the required deployment and monitoring requirements, including a national decision and the relevant regulatory approvals for use. Therefore, in February 2020, the WHO Executive Board urged all Member States to expedite processes for authorizing the importation and use of nOPV2 under EUL [21]. As use of nOPV2 is only permitted as part of an approved outbreak response to cVDPV2, it was not possible to pre-select the countries that will use nOPV2 during the initial use phase.

To address this, WHO and UNICEF regional offices, with the support of GPEI, began the process of preparing all countries at high risk of cVDPV2 for use of nOPV2 in mid 2020, in advance of the EUL being issued. A set of tools and guidance materials to support these preparations were developed across GPEI; these provided clarification on what the readiness requirements are and how to meet them in key domains such as national decision making, regulatory approvals, communications, safety, surveillance, laboratory, vaccine management and outbreak operations. Due to COVID-related restrictions, trainings and reviews were rolled out virtually both for priority countries but also by technical area, such as across the global polio laboratory network, with National Immunization Technical Advisory Groups (NITAGs) and National Regulatory Authorities (NRAs).

Given the large number of countries that began preparing for nOPV2 use at the same time, priority countries were asked to nominate a national focal point for nOPV2 preparations; GPEI provided funding for the deployment of nOPV2 focal points and facilitators to support preparations, where needed. Training and onboarding for these focal points was held virtually, to ensure they were familiar with the tools and guidance, as well as support available to them in their role. The aim over the next few years will be to generate, analyze and use field-use data and information from on-going clinical studies to inform policies on outbreak response, and to strengthen the evidence base in support of full licensure and WHO Prequalification of the vaccine, to transition out of the use of nOPV2 under EUL. Detailed evaluation of genetic characteristics of nOPV2 isolates from sewage and clinical specimens through the global polio laboratory network along with assessment of effectiveness and safety of the vaccine from use in outbreak response in real-world settings would help us determine the impact of the vaccination with nOPV2 in interrupting cVDPV2 transmission.

The first campaigns with nOPV2 were carried out in March 2021 as a response to outbreaks of cVDPV2 in Nigeria and Liberia, with further countries soon following with nOPV2 use.

## 8 Summary

The WHO EUL recommendation for nOPV2 use is a milestone achievement in global health and has paved the way for the accelerated use of unlicensed vaccines during PHEIC including COVID-19 vaccines. Between the initial submission of nOPV2 regulatory dossier to WHO EUL in February 2020, to recommendation in November 2020, an in-depth assessment of pre-clinical, manufacturing, and clinical data on vaccine safety and effectiveness was conducted by an independent expert review committee engaged by the WHO Prequalification team. The multi-pronged approach implemented by the GPEI and vaccine manufacturer PT Bio Farma in coordination with other partner agencies to develop the EUL submission and prepare countries for roll-out of the vaccine is summarised in this paper and provides many lessons for acceleration of clinical trials and manufacturing.

The use of nOPV2 in outbreak response to cVDPV2 is urgently needed due to the demonstrated risk of reseeding through Sabin mOPV2 use. However, the ability to stop outbreaks with nOPV2 is dependent on the implementation of timely, high-quality outbreak response of sufficient scope.

### CRediT authorship contribution statement

**Grace Macklin:** Methodology, Visualization, Writing – original draft, Writing – review & editing. **Corey Peak:** Methodology, Writing – original draft, Writing – review & editing. **Martin Eisenhawer:** Resources, Writing – original draft, Writing – review & editing. **Feyrouz Kurji:** Project administration, Writing – review & editing. **Ondrej Mach:** Writing – original draft, Writing – review & editing. **John Konz:** Investigation, Writing – review & editing. **Chris Gast:** Investigation, Writing – review & editing. **Novilia Sjafri Bachtiar:** Resources, Writing – review & editing. **Ananda S. Bandyopadhyay:** Investigation, Conceptualization, Supervision, Writing – original draft, Writing – review & editing. **Simona Zipursky:** Conceptualization, Supervision, Writing – original draft, Writing – review & editing.

## Declaration of Competing Interest

The authors declare that they have no known competing financial interests or personal relationships that could have appeared to influence the work reported in this paper.
